# Noninvasive scoring system for significant inflammation related to chronic hepatitis B

**DOI:** 10.1038/srep43752

**Published:** 2017-03-10

**Authors:** Mei-Zhu Hong, Linglong Ye, Li-Xin Jin, Yan-Dan Ren, Xiao-Fang Yu, Xiao-Bin Liu, Ru-Mian Zhang, Kuangnan Fang, Jin-Shui Pan

**Affiliations:** 1 Department of Traditional Chinese Medicine, Zhongshan Hospital Xiamen University, Xiamen, 361004 Fujian, China; 2 Graduate Institute of Business Administration, College of Management, Fu Jen Catholic University, New Taipei City, 24205 Taiwan; 3 Department of Gastroenterology, People’s Hospital of Rizhao, Rizhao, 276800 Shandong, China; 4 Department of Gastroenterology, Zhongshan Hospital affiliated to Xiamen University, Xiamen, 361004 Fujian, China; 5 Hepatology Unit and Department of Infectious Diseases, Xiamen Hospital of Traditional Chinese Medicine, Xiamen, 361001 Fujian, China; 6 Department of Infectious Diseases, the First Affiliated Hospital of Xiamen University, Xiamen, 361004 Fujian, China; 7 Department of Statistics, School of Economics, Xiamen University, Xiamen, 361002 Fujian, China

## Abstract

Although a liver stiffness measurement-based model can precisely predict significant intrahepatic inflammation, transient elastography is not commonly available in a primary care center. Additionally, high body mass index and bilirubinemia have notable effects on the accuracy of transient elastography. The present study aimed to create a noninvasive scoring system for the prediction of intrahepatic inflammatory activity related to chronic hepatitis B, without the aid of transient elastography. A total of 396 patients with chronic hepatitis B were enrolled in the present study. Liver biopsies were performed, liver histology was scored using the Scheuer scoring system, and serum markers and liver function were investigated. Inflammatory activity scoring models were constructed for both hepatitis B envelope antigen (+) and hepatitis B envelope antigen (−) patients. The sensitivity, specificity, positive predictive value, negative predictive value, and area under the curve were 86.00%, 84.80%, 62.32%, 95.39%, and 0.9219, respectively, in the hepatitis B envelope antigen (+) group and 91.89%, 89.86%, 70.83%, 97.64%, and 0.9691, respectively, in the hepatitis B envelope antigen (−) group. Significant inflammation related to chronic hepatitis B can be predicted with satisfactory accuracy by using our logistic regression-based scoring system.

Liver biopsy-based histology is critical for prognosis evaluation and decision making with regard to antiviral treatment in patients with chronic hepatitis B (CHB). The key information provided by liver biopsy includes the stage of fibrosis, grade of inflammation, and presence of accompanying diseases. Due to the invasiveness of liver biopsy, not all patients agree to undergo this procedure. For the noninvasive prediction of fibrosis related to chronic hepatitis C (CHC), several scoring systems have been developed, such as the APRI and FIB-4 index^1^,^2^. However, for the prediction of fibrosis related to CHB, the APRI and FIB-4 index might have moderate or even unsatisfactory sensitivity and accuracy^3^,^4^. Transient elastography has been widely used for the assessment of liver fibrosis or cirrhosis with high accuracy through liver stiffness measurement (LSM)^5^,^6^,^7^,^8^. Histological information on fibrosis or cirrhosis can also be achieved through LSM. However, LSM cannot replace liver biopsy currently, as it cannot provide information on intrahepatic inflammation. Our previous study found that the stage of fibrosis is highly correlated with the grade of inflammation^9^, and this finding is supported by another study^10^. Several models have been proposed for the prediction of inflammatory activity related to CHB^10^,^11^,^12^,^13^. Recently, we reported an LSM-based prediction model for inflammatory activity related to CHB, which has high accuracy with balanced sensitivity and specificity^14^.

Although inflammatory activity can be accurately predicted using the LSM-based model, FibroScan cannot be readily introduced in the primary care center. On the other hand, patients with a high body mass index tend to have a high LSM value^15^,^16^. Furthermore, the levels of bilirubin and alanine aminotransferase (ALT) have a significant effect on the accuracy of transient elastography^17^,^18^. Therefore, the present study aims to create a noninvasive scoring system for the prediction of intrahepatic inflammatory activity related to CHB, without the aid of transient elastography.

## Results

### Patient characteristics

Among the 396 enrolled patients, 221 (55.8%) were HBeAg (+) and 175 (44.2%) were HBeAg (−) (Table S1). Both the HBeAg (+) and HBeAg (−) groups had more men than women. The ALT and AST levels were significantly higher in the HBeAg (+) group than in the HBeAg (−) group (*P* < 0.0001). The serum levels of HBV DNA were significantly higher in the HBeAg (+) group than in the HBeAg (−) group for both sets (*P* < 0.0001). Patients in the two sets had similar baseline characteristics. Significant inflammation (G 3 or 4) was noted in 50 (22.6%) and 37 (21.1%) patients from the HBeAg (+) and HBeAg (−) groups, respectively.

### Continuous variables were normalized by z-score normalization

All independent continuous variables, including individual variables and composite variables, were normalized based on z-score normalization. The means and standard deviations of the critical variables are shown in Table S2. The normalized variables are prefixed with “st” (for example, stALT × AST).

### Variables related to significant inflammation with high frequency were screened using LASSO logistic regression

The HBeAg (+) and HBeAg (−) groups were randomly sampled at a ratio of 7:3 100 times. One hundred prediction models based on LASSO logistic regression were constructed for each group. The non-zero variables occurring more than 50 times in the prediction models for the HBeAg (+) and HBeAg (−) groups are shown in Table S3. For the HBeAg (−) group, the most frequently observed variable was CHE/AST (94 times in the 100 prediction models based on LASSO logistic regression), followed by Alb × CHE (90 times), Alb × PreAlb (80 times), and ALT/PreAlb (59 times). For the HBeAg (+) group, the most frequently observed variable was GGT/PLT (82 times), followed by PreAlb × PLT (81 times), Alb × CHE (76 times), and CHE/AST (73 times). In both groups, CHE/AST, Alb × CHE, and GGT/PLT were related to significant inflammation with high frequency.

### Variables with high frequency were further verified using traditional logistic regression

In the HBeAg (−) and HBeAg (+) groups, seven and five variables, respectively, were identified as highly frequent variables (Table S3). The variables with high frequency were further verified using traditional logistic regression on the entire sample of each group. In the HBeAg (−) group, stCHE/AST, stAlb × CHE, stAlb × PreAlb, and stGGT/PLT were found to have a strong effect on the prediction of significant inflammation (Table 1). In the HBeAg (+) group, stCHE/AST, stAlb × CHE, stGGT/PLT, and stPreAlb × PLT were found to have a strong effect on the prediction of significant inflammation (Table 1).

### Construction of the prediction models

The final prediction models for each group were constructed based on the coefficients shown in Table 1, using traditional logistic regression, and were as follows:

For the HBeAg (−) group, prediction model was constructed:


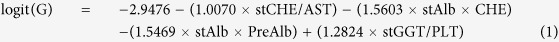


For the HBeAg (+) group, prediction model was constructed:


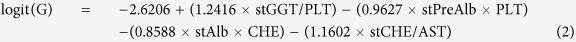


### Diagnostic performance of the prediction model for significant inflammation

**Figure 1:**
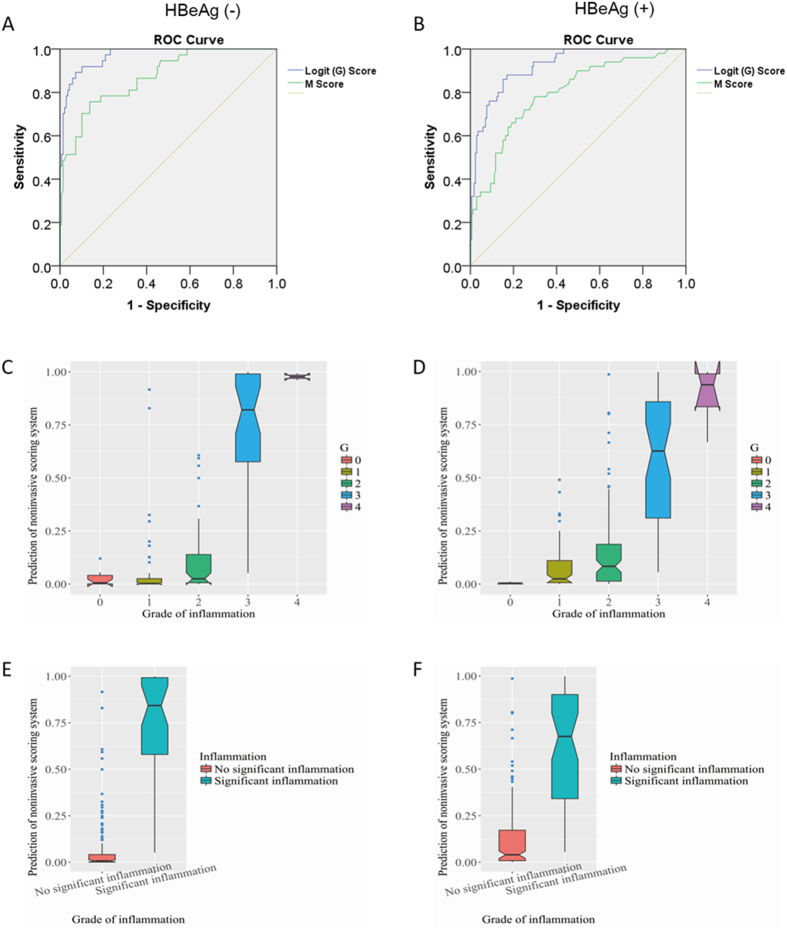
Receiver operating characteristic (ROC) curve of our traditional logistic regression-based score (logit [G] score), Mohamadnejad’s score (M score), and the logit (G) score for differentiating significant inflammation. (**A**) ROC curve of the logit (G) score and M score for HBeAg (−) patients; (**B**) ROC curve of the logit (G) score and M score for HBeAg (+) patients; (**C**) The mean probabilities of predicting patients with significant inflammation (G 3, 4) increased with the increase in the grade of inflammation in the HBeAg (−) group; (**D**) The mean probabilities of predicting patients with significant inflammation (G 3, 4) increased with the increase in the grade of inflammation in the HBeAg (+) group. (**E**) The mean probabilities of predicting patients with significant inflammation are significantly higher in the patients with significant inflammation (G 3, 4) in the HBeAg (−) group; (**D**) The mean probabilities of predicting patients with significant inflammation are significantly higher in the patients with significant inflammation (G 3, 4) in the HBeAg (+) group.

The AUC, sensitivity, and specificity were 0.9691, 91.89%, and 89.86%, respectively, in the HBeAg (−) group and were 0.9219, 86.00%, and 84.80%, respectively in the HBeAg (+) group (Table 2). Cut-off values of 0.2163 for the HBeAg (−) group and 0.2271 for the HBeAg (+) group were identified for the diagnosis of significant inflammation. Additionally, the PPV and NPV for both groups were appropriate. The AUCs of the prediction models are shown in Fig. 1A,B. In both the HBeAg (+) and HBeAg (−) groups, there was a significantly higher probability of predicting patients with significant inflammation (G 3 and 4) than without inflammation (G 0) or with mild (G 1) or moderate inflammation (G 2) (Kruskal-Wallis test, *P* < 0.0001) (Fig. 1C,D). If the enrolled patients were divided into two groups, group without significant inflammation (G 0, 1 and 2) and group with significant inflammation (G 3 and 4), probability of predicted significant inflammation was significantly higher in the group with significant inflammation (G 3 and 4) (Kruskal-Wallis test, *P* < 0.0001) (Fig. 1E,F).

### Diagnostic performance of another model

Mohamadnejad *et al*.^12^ reported a prediction model for significant inflammation, involving age, HBV DNA level, AST, and albumin. Using our data, the AUCs according to this previous model were 0.7970 and 0.8720 in the HBeAg (+) and HBeAg (−) groups, respectively (Table 2). Additionally, the PPV and NPV for the HBeAg (+) and HBeAg (−) groups according to this previous model were 0.4318 and 0.9098, and 0.6410 and 0.9118, respectively (Table 2). The diagnostic performance of our model was notably better than that of the model by Mohamadnejad *et al*. (Table 2).

## Discussion

We constructed a predictive model based on serum markers for significant inflammation related to CHB. The most distinguishing feature of our scoring system is that several combined variables were used as independent variables for the prediction of inflammation. Additionally, the diagnostic performance of our scoring algorithm was excellent for both HBeAg (+) and HBeAg (−) patients. Moreover, the scoring system was feasible and easy to use. A web-based scoring system has been developed based on our prediction model (http://iyves.me:3838/fkn/).

In recent years, LASSO logistic regression and MCP logistic regression have been proposed for the assessment of data with large number of independent variables^19^,^20^. In the pretreatment process, we randomly divided the data into a training set and a validation set in the ratio of 7:3 100 times. Overall, the LASSO and MCP logistic regression models achieved better prediction performance with regard to the validation set than traditional logistic regression models (Table S4 and Fig. S1). As shown in Table S4, all indices of prediction performance were high in the training set, while some indices had obvious reductions in the validation set, such as sensitivity, PPV, and AUC. It has been indicated that the traditional logistic regression models had the problem of over-fitting. Moreover, the LASSO tended to perform better than the MCP. Due to the diagnostic performance, LASSO has been widely used in the field of medicine or related subjects^21^,^22^,^23^.

Overall, the verification of independent variables in the present research can be divided into two stages. First, we constructed a “pool” of all candidate independent variables, including individual variables and combined variables, which involved the multiplication or division of two individual variables, followed by LASSO logistic regression-based screening of independent variables with high frequency. Second, the non-zero variables with high frequency in the prediction models based on LASSO logistic regression were further verified using traditional logistic regression on the entire sample of each group. Of interest, as shown in Table 1, in both the HBeAg (−) and HBeAg (+) groups, the verified independent variables were all combined variables, which included CHE, AST, Alb, PreAlb, GGT, and PLT. Theoretically, the multiplication or division of two variables would gain more powerful discernibility than any one of the included variables. It has been indicated in our previous research that patients with significant inflammation tended to have higher AST and GGT levels, and have lower CHE, Alb, and PreAlb levels^14^. As was shown in the research by Pan *et al*.,^24^ PLT was significantly negatively related to the degree of liver inflammation, which was in accordance with the finding by Shoaei *et al*.^25^. Research has indicated that PLT involves intrahepatic inflammation via mediation of cytotoxic T lymphocyte-induced liver damage^26^. PLT-neutrophil interaction in a Toll-like receptor 4-dependent pattern would lead to robust neutrophil activation^27^. Thus, it appears reasonable that PLT is correlated with intrahepatic inflammation.

In our scoring system, intrahepatic inflammation was divided into no inflammation to moderate inflammation (G 0–2) and severe inflammation (G 3–4). Although the dependent variable was a dichotomy index, our scoring system has a satisfactory ability to differentiate the five scales of intrahepatic inflammation according to the Scheuer system. In the HBeAg (−) group, the probability that patients would be predicted to have significant inflammation (G 3, 4) increased with the grade of inflammation. Similar phenomena were observed in the HBeAg (+) group (Fig. 1C,D). The model by Mohamadnejad *et al*.^12^ can predict significant inflammation (G 3 and 4) with AUCs of 0.872 and 0.797 in HBeAg (−) and HBeAg (+) patients, respectively. The sensitivity, specificity, PPV, and NPV of our model were better than those of the model by Mohamadnejad *et al*. for both HBeAg (+) and HBeAg (−) patients, and therefore, our model is more applicable in clinical practice (Fig. 1A,B).

In our previous study, we reported an LSM-based model for significant inflammation, which had nearly perfect AUCs of 0.971 and 0.977 in the HBeAg (+) and HBeAg (−) patients, respectively^14^. If FibroScan is available, significant inflammation can be precisely predicted. However, FibroScan is not commonly present in the primary care center. Additionally, it cannot be neglected that high body mass index, high bilirubinemia, and high level of ALT have a noted effect on the accuracy of transient elastography. Thus, it was necessary to develop a complementary scoring system that can be employed in the absence of transient elastography.

In conclusion, significant inflammation related to CHB can be predicted with satisfactory accuracy by using our logistic regression-based scoring system.

## Patients and Methods

### Patients

Treatment-naïve CHB patients referred to Chenggong Hospital Xiamen University, Zhongshan Hospital Xiamen University, and Xiamen Hospital of Traditional Chinese Medicine were enrolled in this study. The present study was approved by the ethics committees of Chenggong Hospital Xiamen University, Zhongshan Hospital Xiamen University, and Xiamen Hospital of Traditional Chinese Medicine, and was conducted according to the principles of the Declaration of Helsinki. Written informed consent was obtained from all participants prior to liver biopsy and study enrollment. No identifying information or image was included in the present research. Liver biopsies were performed on the day of serum sample collection or within 2 days of sample collection. Significant inflammation was defined as Grade 3 or 4 inflammation according to the Scheuer scoring system^28^.

Participants were enrolled according to the status of hepatitis B surface antigen (HBsAg). The inclusion criterion was HBsAg positivity with scheduled liver biopsy, regardless of hepatitis B envelope antigen (HBeAg) status. The exclusion criteria were co-infection of hepatitis C virus, hepatitis D virus, and human immunodeficiency virus; malnutrition; significant steatosis; alcoholic fatty liver; and decompensated cirrhosis. A total of 396 consecutive treatment-naïve CHB patients were eventually retrospectively analyzed. The patients were divided randomly into a training set and a validation set (ratio, 7:3). The training set was used to construct the prediction model, whereas the validation set was used to evaluate the efficiency of the prediction model. In order to obtain a stable prediction efficiency, the following cycle was repeated 100 times: random grouping (ratio, 7:3), construction of the prediction model based on the training set, and finally, verification of the prediction efficiency using the validation set.

### Diagnostic tests

Hepatitis B virus (HBV) DNA levels were detected using quantitative fluorescence polymerase chain reaction with a lower limit of detection of 500 IU/mL. For the construction of the prediction model, HBV DNA was expressed as log_10_ IU/mL, whereas serum ALT and aspartate aminotransferase (AST) levels were expressed as IU/L. Albumin (ALB), γ-glutamyl transpeptidase (GGT), cholinesterase (CHE), globulin (GLB), and pre-albumin (Pre-ALB) were assessed using the chemistry analyzer TBA-120FR (Toshiba, Tochigi, Japan). Liver histology was evaluated by two independent pathologists who were blinded to the study design. Histological scores were calculated and confirmed by a panel of pathologists in case of disagreement between the two pathologists with regard to the pathological diagnosis. Patients were divided into two groups according to the Scheuer scale (G 0, 1, and 2, and G 3 and 4)^14^.

### Data analysis

Statistical analyses were performed using R version 3.2.3^29^. The 396 patients were classified into the following two groups according to the status of HBeAg: HBeAg (−) (n = 175) and HBeAg (+) (n = 221). For each patient, potential independent variables included individual variables, such as ALB, ALT, AST, CHE, GGT, GLB, Pre-ALB, platelet (PLT), prothrombin time (PT), and HBV DNA, and composite variables, such as ALT × AST and ALT/ALB. All potential independent variables, including individual variables and composite variables, were screened for critical variables contributing to the response. Continuous variables on different scales were normalized based on z-score normalization. The normalized variables were calculated using the following formula:


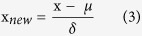


A logistic regression model and least absolute shrinkage and selection operator (LASSO) logistic regression model were adopted to compare the performance of the prediction models and to screen critical independent factors for each group^19^. The methodologies were implemented in the R package stats version 3.2.3, glmnet version 2.0–5, and ncvreg version 3.5-1^29^,^30^,^31^. The regularization parameter lambda values of the LASSO were evaluated by 50-fold cross-validation.

The receiver operating characteristic (ROC) curve and the area under the curve (AUC) were calculated to evaluate the accuracy of all prediction models. The optimal cut-off value of the training set was determined by the closest-to-(0,1) criterion, i.e., the minimum value of


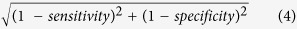


which was the point on the ROC curve closest to the top left part of the plot with perfect sensitivity or specificity^32^,^33^. The specificity, sensitivity, accuracy, positive predictive value (PPV), and negative predictive value (NPV) were also determined. The means and 95% confidence intervals (CIs) of these measurements were obtained by repeating the above process 100 times. The frequency of non-zero coefficients was computed to identify critical variables contributing to the response, and the final logistic regression prediction models for the two groups were fitted on those identified critical independent variables.

## Additional Information

**How to cite this article:** Hong, M.-Z. *et al*. Noninvasive scoring system for significant inflammation related to chronic hepatitis B. *Sci. Rep.* **7**, 43752; doi: 10.1038/srep43752 (2017).

**Publisher's note:** Springer Nature remains neutral with regard to jurisdictional claims in published maps and institutional affiliations.

## Supplementary Material

Supplementary Information

## Figures and Tables

**Table 1:** Critical variables further verified by traditional logistic regression.

HBeAg (−)			HBeAg (+)		
Variable	Coefficient	*P* value	Variable	Coefficient	*P* value
Intercept	−2.9476	<0.01	Intercept	−2.6206	<0.01
stCHE/AST*	−1.0070	0.05	stGGT/PLT*	1.2416	<0.01
stAlb × CHE*	−1.5603	0.01	stPreAlb × PLT*	−0.9627	0.02
stAlb × PreAlb*	−1.5469	0.02	stAlb × CHE*	−0.8588	0.02
stGGT/PLT*	1.2824	0.03	stCHE/AST*	−1.1602	0.01

^*^z-score normalized variable.

**Table 2:** Diagnostic performance of the final logistic regression model and other model.

Index	Our Score		Mohamadnejad’s Score	
	HBeAg (−)	HBeAg (+)	HBeAg (−)	HBeAg (+)
Cut-off	0.2163	0.2271	4.6500	1.5900
Sensitivity (%)	91.89	86.00	67.57	76.00
Specificity (%)	89.86	84.80	89.86	70.76
Accuracy (%)	90.29	85.07	85.14	71.95
PPV (%)	70.83	62.32	64.10	43.18
NPV (%)	97.64	95.39	91.18	90.98
AUC	0.9691	0.9219	0.8720	0.7970

PPV, positive predictive value; NPV, negative predictive value; AUC, area under curve.
