# Supplementary material for: Noninvasive scoring system for significant inflammation related to chronic hepatitis B
Source: Sci Rep. 2017 Mar 10;7:43752. doi: 10.1038/srep43752 (PMC5345042; doi:10.1038/srep43752)
Supplement: Supplementary Information [file srep43752-s1.pdf]

Noninvasive scoring system for significant inflammation related to chronic hepatitis B

Mei-Zhu Hong[1, †], Linglong Ye[2, †], Li-Xin Jin[3, †], Yan-Dan Ren[4], Xiao-Fang Yu[5], Xiao-Bin Liu[6], Ru-Mian Zhang[5], Kuangnan Fang[7, *], Jin-Shui Pan[4, *]

Table S1. Characteristics of the enrolled patients

	HBeAg (+)<br>(n = 221)	HBeAg (-)<br>(n = 175)
Age, yr (Median)	31.0	40.0
Male, n (%)	165 (74.7)	144 (82.3)
ALT, IU/L (Median)	97.0	61.0
Normal ALT, n (%)	23 (10.4)	45 (25.7)
AST, IU/L (Median)	50.0	37.0
Normal AST, n (%)	80 (36.2)	96 (54.9)
CHE, IU/L	8109 ± 1664	8423 ± 1861
GGT, IU/L (Median)	37.0	36.0
Globulin, g/L	28.9 ± 4.3 (Mean)	28.0 (Median)
Albumin, g/L (Median)	44.2	44.1
Pre-Albumin, mg/L	211.0 ± 63.0	230.2 ± 69.7
Platelet, 10^9/L	193.7 ± 50.4	184.4 ± 50.1
**Liver histology***		
Inflammation activity, n (%)		
G0	7 (3.2)	13 (7.4)
G1	71 (32.1)	68 (38.9)
G2	93 (42.1)	57 (32.6)
G3	46 (20.8)	35 (20.0)
G4	4 (1.8)	2 (1.1)

*According to the Scheuer scoring system.

ALT, alanine aminotransferase; AST, aspartate aminotransferase; CHE, cholinesterase; GGT, γ-glutamyl transpeptidase

Table S2. The means and standard deviations of critical variables

	GGT/PLT	Alb×CHE	Alb×PreAlb	PreAlb×PLT	CHE/AST
Mean (μ)	0.3405	366.5261	9783.6872	42701.4965	0.2060
Standard deviation (σ)	0.4751	93.7852	3358.0024	19429.0485	0.1358

GGT, γ-glutamyl transpeptidase; PLT, platelet; Alb, albumin; CHE, cholinesterase; PreAlb, Pre-Albumin; AST, aspartate aminotransferase.

Table S3 The nonzero coefficients with more than 50 times in LASSO logistic regression

HBeAg (-)		HBeAg (+)	
Variables	Times	Variables	Times
CHE/AST	94	GGT/PLT	82
Alb×CHE	90	PreAlb×PLT	81
Alb×PreAlb	80	Alb×CHE	76
APRI	76	CHE/AST	73
ALT/PreAlb	59	CHE×PLT	53
GGT/PLT	57		
AST/PLT	51		

Table S4 Prediction performance of LASSO logistic regression, MCP logistic regression, and traditional logistic regression models

		HBeAg (-)		HBeAg (+)	
		Training set	Validation set	Training set	Validation set
LASSO	Lambda	0.0623	0.0623	0.0486	0.0486
	Cut-off	0.2466	0.2466	0.2401	0.2401
	Specificity (%)	91.27	88.71	86.14	84.11
	Sensitivity (%)	91.22	81.94	87.31	78.55
	Accuracy (%)	91.26	87.27	86.41	82.85
	PPV (%)	74.39	67.17	64.88	59.80
	NPV (%)	97.46	95.07	95.89	93.13
	AUC	0.9691	0.9406	0.9283	0.9022
MCP logistic regression	Lambda	0.0335	0.0335	0.0373	0.0373
	Cut-off	0.2546	0.2546	0.2378	0.2378
	Specificity (%)	90.64	88.48	85.33	82.84
	Sensitivity (%)	91.24	78.00	86.09	74.32
	Accuracy (%)	90.77	86.17	85.50	80.86
	PPV (%)	73.30	65.01	63.59	56.85
	NPV (%)	97.45	93.90	95.47	91.75
	AUC	0.9627	0.9164	0.9216	0.8798
Traditional	Cut-off	0.5000	0.5000	0.4814	0.4814

logistic	Specificity (%)	99.18	86.29	95.02	84.78
regression	Sensitivity (%)	97.09	51.19	84.66	52.72
	Accuracy (%)	98.73	78.75	92.66	77.41
	PPV (%)	97.21	50.06	84.15	51.66
	NPV (%)	99.20	86.99	95.52	85.86
	AUC	0.9814	0.6906	0.9021	0.6947

PPV, positive predictive value; NPV, negative predictive value; AUC, area under curve

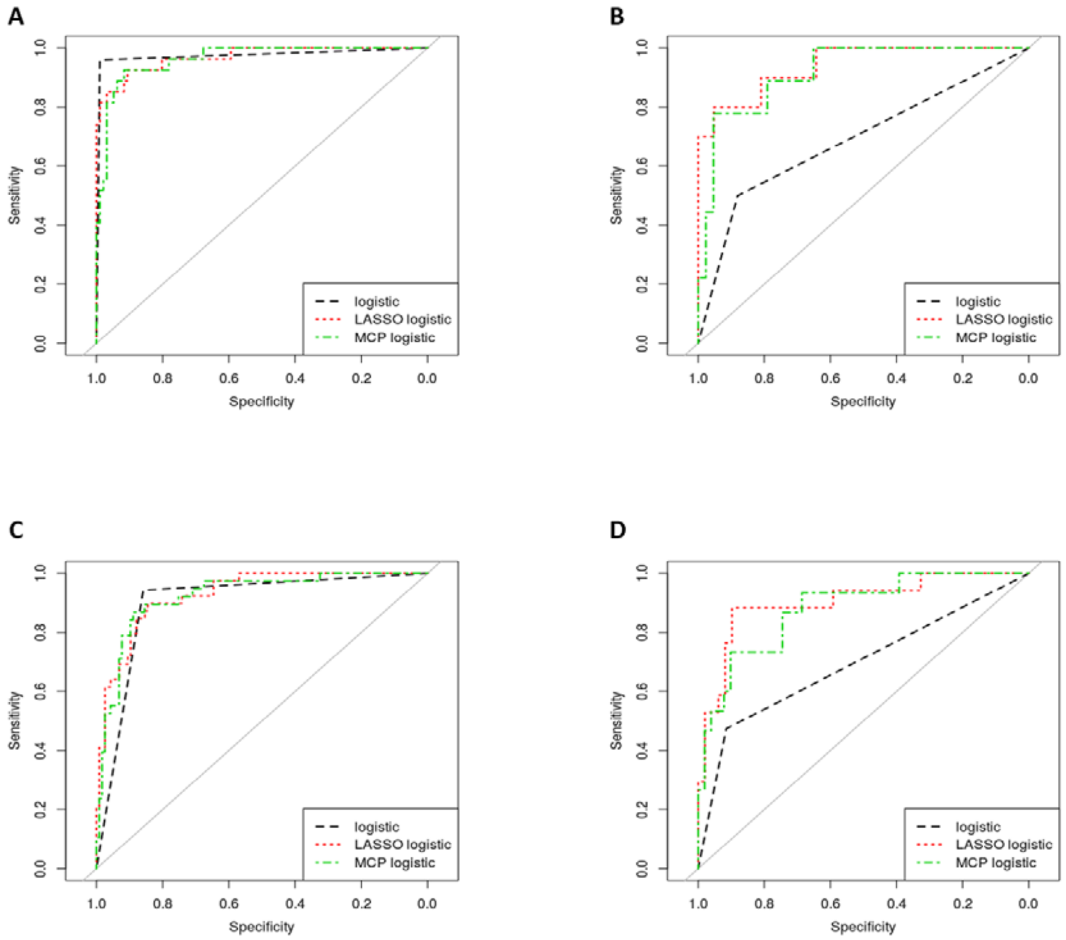

Fig. S1. Receiver operating characteristic (ROC) curve of the traditional logistic regression-based model (logistic), LASSO logistic regression model (LASSO logistic), and MCP logistic regression model. (A) ROC curves of the three models for the training set among HBeAg (-) patients; (B) ROC curves of the three models for the validation set among HBeAg (-) patients; (C) ROC curves of the three models for the training set among HBeAg (+) patients; (D) ROC curves of the three models for the validation set among HBeAg (+) patients.
